# Public-private partnerships and the politics of alcohol policy in England: the Coalition Government’s Public Health ‘Responsibility Deal’

**DOI:** 10.1186/s12889-019-7787-9

**Published:** 2019-11-08

**Authors:** Benjamin Hawkins, Jim McCambridge

**Affiliations:** 1 Faculty fo Public Health and Policy, London School of Hygiene and Tropical Medicine, 15-17 Tavistock Plave, London, WC1H9SH UK; 2 Department of Health Sciences, University of York, Heslington, York, YO10 5DD UK

**Keywords:** Responsibility Deal, Alcohol policy, Alcohol industry, Corporations, Self-regulation, Co-regulation, UK

## Abstract

**Background:**

The 2010–2015 Conservative-led Coalition Government launched their flagship Public Health Responsibility Deal (PHRD) for England in 2011; a year before their alcohol strategy. This co-regulatory regime placed alcohol industry actors at the heart of policy-making, but was viewed with scepticism by public health actors. This article examines the ways in which the PHRD structured the alcohol policy environment throughout this period, which included the rejection of evidence-based policies such as minimum unit pricing.

**Methods:**

This article draws on 26 semi-structured interviews with policy actors (parliamentarians, civil servants, civil society actors and academics) in 2018. Respondents were identified and recruited using purposive sampling. Interviews were recorded, transcribed and analysed using thematic coding.

**Results:**

The PHRD shaped the context of alcohol policy development at Westminster throughout this period. It circumscribed the policy space by taking evidence-based measures not amenable to industry partnership off the agenda. While the PHRD created important opportunities for industry engagement with policy-makers, it undermined public health actors’ access to government, particularly following their withdrawal from the process. Moreover, the PHRD demonstrates the enduring appeal of partnership as a policy idea for governments, despite a lack of evidence of their effectiveness.

**Conclusions:**

This study of the PHRD demonstrates the ways in which industry actors are able to influence policy through long-term relationship building and partnership working on policy decision-making. Whilst such partnership approaches may appear to have the potential to mitigate some of alcohol harms, they create fundamental conflicts of interest, and may undermine the very causes they seek to further.

## Background

This article examines the Conservative-Liberal Democrat Coalition Government’s Public Health Responsibility Deal (PHRD) [1] and the structuring effects this had on UK alcohol policy debates in the period between 2010 and 2015. The origins of the PHRD can be traced back to the period before the 2010 general election at which the Coalition Government came to power. Concerns about rising burden of obesity and non-communicable diseases (NCDs) such as cancer, heart disease, hypotension and stroke increased pressure for government action to tackle alcohol consumption, poor diet and lack of exercise associated with these conditions. In response to this, the Conservative Party’s health lead, Andrew Lansley, convened a *Public Health Commission* (PHC) involving leading companies in the food, alcohol, retail and fitness industries in 2008 [2]. The PHC was chaired by Dave Lewis of Unilever, which also provided the premises and the secretariat for the Commission’s meetings.

In the same year, the Scottish Government published its draft alcohol strategy, which included a consultation on introducing minimum unit pricing (MUP) for alcohol [3]. Pricing measures such as MUP are strongly supported by international research evidence on policies to address alcohol related harms [4], but opposed by the alcohol industry, which favours instead voluntary and self- and co-regulatory approaches such as the PHRD [5]. Co-regulatory regimes refer to institutionalised engagement between government and industry actors to deliver policy goals such as the PHRD. Self-regulatory regimes permit industry bodies to regulate their activities with minimal government intervention or oversight, and include activities such as the Portman Group’s code of practice on the marketing of alcohol products. While some of the policy studies and public administration literature suggests that self- and co-regulation may be advantageous [6, 7], their adoption in the field of alcohol (and health policy more generally) has been widely criticised by public health actors. Both self- and co-regulatory regimes have limited evidence of their effectiveness [4] and, it is argued, create unavoidable conflicts of interests (COI) between the profit motives of alcohol companies and the goal of protecting public health [8, 9]. Moreover, government involvement confers legitimacy on the corporate social responsibility (CSR) work of industry actors in ways which promote their corporate interests without addressing health needs.

The Scottish alcohol strategy represented a sea change in UK alcohol policy, which had heretofore adhered to an industry favourable agenda [10]. This followed successful efforts by health advocates to reframe policy debates in terms of evidence-based, whole population interventions [5, 11], and vital preparatory work on MUP undertaken within the Department of Health (DH) in London [12], such as commissioning the first modelling of the effects of alcohol pricing by researchers at the University of Sheffield. Following a six-year delay in implementation after the MUP legislation was passed by the Scottish Parliament, as a result of industry legal challenges [13, 14], MUP entered into force in Scotland in May 2018. Having been included in the government’s 2012 alcohol strategy [15], plans to introduce MUP in England have been stalled since 2013 with no end to the hiatus in sight at the time of writing [16].

Previous studies have evaluated the effectiveness of the PHRD as a public health intervention and the success of participants in meeting its objectives [17–19]. The aim of the current article is to examine the *political* consequences – as opposed to population health effects – of the PHRD’s Responsibility Deal Alcohol Network (RDAN) within the wider context of UK alcohol policy debates since 2010. In so doing, we identify the role, which co-regulatory regimes such as the PHRD can play within the alcohol industry’s political strategies and the effects these may have on the development of public health policies. As the key alcohol policy controversy in this period, MUP provides essential context for the policy developments analysed here [20]. This study will, therefore, examine attempts by industry and other actors to link the PHRD to the issue of MUP, and how far this may have been relevant to the reversal of the Government’s commitment to introduce the policy in England [21–24].

Whilst our focus is on the alcohol industry and UK alcohol policy, similarities have been identified between industry strategies in other sectors including the tobacco and pharmaceutical industry [25–28]. As such, the analysis presented here is of wider relevance to understanding the impact of co-regulatory regimes in structuring health policy debates and favouring vested interests in other policy areas and policy-making contexts.

### The PHRD: an overview

The PHRD is a co-regulatory regime designed to bring together industry actors, public health NGOs, medical associations and other concerned parties (e.g. the Police) with policymakers to work towards improving public health through a series of agreed activities (see Table 1). The PHRD included, amongst others, a specific alcohol network made up of government, public health and alcohol industry participants, including the UK’s largest supermarket chains [1]. Six public health bodies – Alcohol Concern, The British Association for the Study of the Liver, The British Liver Trust, The British Medical Association, The Institute of Alcohol Studies and The Royal College of Physicians – who had been involved in the initial discussions around the formation of the RDAN refused to sign up to the agreement, citing conflicts of interest arising from alcohol industry involvement, the focus on weak, industry favourable policy approaches and the lack of clarity about enforcement mechanisms should industry fail to meet their commitments [29].

**Table 1:** The Responsibility Deal Alcohol Network Pledges

Pledge	Details
A1.	We will ensure that over 80% of products on shelf (by December 2013) will have labels with clear unit content, NHS guidelines and a warning about drinking when pregnant.
A2.	We will provide simple and consistent information in the on-trade (e.g. pubs and clubs), to raise awareness of the unit content of alcoholic drinks, and we will also explore together with health bodies how messages around drinking guidelines and the associated health harms might be communicated.
A3.	We will provide simple and consistent information as appropriate in the off-trade (supermarkets and off-licences) as well as other marketing channels (e.g. in-store magazines), to raise awareness of the units, calorie content of alcoholic drinks, NHS drinking guidelines, and the health harms associated with exceeding guidelines.
A4.	We commit to ensuring effective action is taken in all premises to reduce and prevent under-age sales of alcohol (primarily through rigorous application of Challenge 21 and Challenge 25).
A5.	We commit to maintaining the levels of financial support and in-kind funding for Drinkaware and the “Why let the Good times go bad?” campaign as set out in the Memoranda of Understanding between Industry, Government and Drinkaware.
A6.	We commit to further action on advertising and marketing, namely the development of a new sponsorship code requiring the promotion of responsible drinking, not putting alcohol adverts on outdoor poster sites within 100 m of schools and adhering to the Drinkaware brand guidelines to ensure clear and consistent usage.
A7(a).	In local communities we will provide support for schemes appropriate for local areas that wish to use them to address issues around social and health harms, and will act together to improve joined up working between such schemes operating in local areas as: ▪ Best Bar None and Pubwatch, which set standards for on-trade premises ▪ Purple Flag which make awards to safe, consumer friendly areas ▪ Community Alcohol Partnerships, which currently support local partnership working to address issues such as under-age sales and alcohol related crime, are to be extended to work with health and education partners in local Government ▪ Business Improvement Districts, which can improve the local commercial environment.
A7(b).	To support our pledge to provide schemes appropriate for local areas that wish to use them to address issues around social and health harms, we will fund and/or support industry action in Local Alcohol Action Areas, by ensuring that suitable existing partnership schemes are in the process of being rolled out in Local Alcohol Action Areas by March 2015.
A8(a).	As part of action to reduce the number of people drinking above the guidelines, we have already signed up to a core commitment to “foster a culture of responsible drinking which will help people drink within guidelines”. To support this we will remove 1bn units of alcohol sold annually from the market by December 2015, principally through improving consumer choice of lower alcohol products.
A8(b).	To support our pledge to remove a billion units of alcohol sold annually from the market, we will carry out a review of the alcohol content and container sizes of all alcohol products in our portfolio. By December 2014 we will not produce or sell any carbonated product with more than (4) units of alcohol in a single-serve can.
A9.	We will financially support the Lifeskills Education and Alcohol Foundation (LEAF) with a minimum of £250,000 as a start-up fund. Subject to favourable reporting and evaluation of delivery, we will seek to increase programme scope through funding from the alcohol industry and others.

The RDAN was chaired jointly by Prof. Mark Bellis from the Faculty of Public Health and Jeremy Beadles, the Chief Executive of the Wine and Spirit Trade Association, with ministerial oversight provided by Paul Burstow MP, Minister of State for Care Services at the Department of Health (DH) Services. Jeremy Beadles was replaced by Henry Ashworth, Chief Executive of the Portman Group in February 2012. Prof. Nick Sheron replaced Mark Bellis as Co-Chair in November that year [22]. Tensions between industry and public health actors came to a head once more in the summer of 2013, following the announcement by the UK Government that it would not be proceeding with its plan to introduce MUP for alcohol in England. This led to the high profile resignation of the remaining public health bodies, including Cancer Research UK, Alcohol Research UK, the Faculty of Public Health and the UK Health Forum leaving only two health organisations [30]; both with financial ties to the alcohol industry (Addaction and Mentor UK) on board [31]. With the departure of almost all the non-governmental public health bodies the RDAN was fatally wounded although it did continue to meet during 2014 in the hope that the departed actors may be convinced to return. It was formally disbanded in 2015 with limited publicity.

The RDAN centred on a list of pledges made by the industry, apparently designed to reduce the harms arising from alcohol consumption. In keeping with industry actors’ policy preferences [32], and wider corporate social responsibility activities [33], the pledges reflect a largely individual, versus population-level, framing of alcohol-related harm and how it may be reduced. Population measures are found by the existing research literature to be the most effective means of reducing harms [4], but are opposed by industry because – in seeking aggregate, population-level reductions in consumption – as they threaten to reduce sales and profits. The highest profile initiatives emerging from the of the RDAN centred on alcohol labelling (A1) and a commitment by the industry to remove ‘a billion units’ of alcohol from the market on a voluntary basis as a result of product reformulations and the introduction of lower alcohol products (A8).

The PHRD was subject to an independent evaluation, undertaken by researchers at the London School of Hygiene and Topical Medicine [19]. This concluded that the pledges had had limited impact on consumption and harms and that in many cases focussed on activities already being undertaken by the industry, rather than on additional measures [18]. The evaluation also found also that commitments on product labelling (A1) and the ‘billion unit pledge’ (A8) had not been fully achieved, whilst others lacked clearly time-limited and measurable deliverables (A2, A3, A6) and thus were thus not amenable to accurate evaluation [18].

## Methods

This article draws on 26 semi-structured interviews undertaken by the first author between February and October 2018 with civil servants and government actors (*n* = 8) from relevant ministries and agencies, members of the U.K. Parliament (*n* = 1) and the Scottish Parliament (*n* = 1) and civil society actors (from alcohol related NGOs, medical associations and public health bodies) (*n* = 13) and academic researchers (*n* = 3) in London and Edinburgh [34, 35]. Where interviewees fell within more than one category they were classified according the role through which they engaged with the PHRD and were thus identified as respondents for this study. Interviewees were initially identified and recruited using purposive sampling based on earlier analyses of the UK alcohol policy context [5, 13, 14, 20, 36–38] and through examining relevant documents and websites (e.g. those relating to the PHRD and RDAN) to undertake a preliminary stakeholder mapping [39, 40].

We decided not to interview industry actors in at the outset, departing from the approach used in our earlier interview study of UK alcohol policy. This decision was taken for a number of reasons including uncertainties about access and the additional complexity anticipated in the data in light of our previous findings, which exposed industry actors in ways they would prefer not to be represented (whereas previously we were unknown to industry actors). An important implication of this decision is that the dataset is restricted to perceptions of industry actors as held by other actors. Nonetheless, we contend that it is possible to understand the dynamics of the PHRD through triangulation of perspectives from interviewees from different sectors, including those who had engaged with and worked closely with industry actors. In addition, snowball sampling was used whereby interviewees were asked to suggest further respondents. These responses, alongside the data generated by interviews, were used to assess when data saturation had been reached.

Respondents were contacted via email and by phone and interviews were undertaken at a place of their convenience, usually their places of work, in keeping with the ethics approval granted by the University of York. Interviews were audio-recorded and transcribed, and were semi-structured following a protocol developed by both authors in advance (available to researchers on request). As this article emerges from a wider study of UK alcohol policy, interviews covered the PHRD and other key developments in UK alcohol policy since 2010. While the same topics were explored with all respondents, questions were adapted to interviewees from different settings and different sectors (i.e. specific questions were asked of policy makers from different ministries or of other types of actor to focus on their specific policy expertise and involvements in the issues being discussed). Finally, such interviews require the flexibility to explore and probe topics and themes which emerge in situ but may not have been foreseen in advance. Interviews were conducted by a reflexive practitioner highly familiar with the use of this method to study controversial topics, whilst making interviewees consciously aware of their and our positionality in the research process [41].

Thematic analysis of the transcripts was led by the first author in liaison with the second author, based on the 6 phases of thematic analysis identified by Braun and Clarke and tailored to the specific requirements of the subject matter of the current study [42]. Transcripts were first reviewed by the first author electronically as word documents as the first stage in a process of analysis. Parts of the text which required further clarification through reference to the interview recording, or which required confirmation through references to outside sources (e.g. relevant policy documents to confirm dates and sequences of events), were noted with comment boxes. Relevant sections of the transcripts which related to important events, processes, concepts or themes were coded in the text using the highlighting tool and were recorded in a separate themes document created to summarise and order the emerging themes [35], usually as a paraphrased summary or precis of the relevant section of the interview transcript, with direct quotations also copied and pasted. As subsequent transcripts were reviewed these were noted as additional examples of existing themes or new themes were added and relevant themes and categories were also modified or merged to take account of additional information [35].

While the initial thematic analysis was being undertaken by the first author the transcripts were reviewed ‘blind’ by the second author, who summarised and noted independently key information and themes emerging from each interview. These parallel analyses of the interviews formed the basis of a series of discussions between authors which refined the key themes. The presentation below is organised thematically and reflects the key issues relating to the PHRD and RDAN which arose out of the data examined. The first draft of the article was written by the first author and worked on by the second across several iterations of the draft. Interview based studies such as these depend on the accounts of respondents and, as such, reflect the experiences and perspectives of respondents. While the principle of triangulation [43] seeks to mitigate bias and provide as full an account of events as possible, these are necessarily circumscribed by the range of respondents and other data sources available.

## Results

This is a study of the political consequences of the PHRD. There is an explicitly temporal component to the narrative organisation of the material in the analysis that follows, as the order in which events surrounding the PHRD unfolded was key in determining the development of the wider alcohol policy context including in connection with MUP. Events prior to the formation of the Coalition Government, and the early adoption of the PHRD by the new government, structured the content of the subsequent alcohol strategy, with implications for moves towards adopting MUP.

### Shaping the ideational context of alcohol policy development

The objective for Andrew Lansley’s PHC was to develop partnership-based responses to NCDs involving key industry actors, in keeping with the wider, market-focussed ideological orientation of his party, which could then be translated into policy following the entry of the Conservatives to government. Asked how the PHC was translated into policy in the form of the PHRD following the 2010 general election, a government respondent commented:

It seemed to happen quite seamlessly and quickly, really, quite soon after the coalition took power, and then the responsibility deal took shape so there was a big responsibility deal meeting which oversaw the initiative and then there were separate groups on alcohol, obesity and so on. It didn’t cover tobacco. So really it was kind of an example where what they’ve said in opposition was really what started to happen, and obviously there was a lot more detail to get into once they were in government.

The introduction of the PHRD in March 2011 predated by a year the publication of the government’s alcohol strategy [15]. The latter included a commitment to introduce MUP in England, alongside a range of other, more industry favourable, policy measures in keeping with the preceding policy regime [44]. The details of the billion unit pledge were announced on 23 March 2012; the very same day that the Government’s alcohol strategy – encompassing the MUP commitment – was published, although the pledge was not at all referred to in the strategy itself.

The temporal sequencing of these alcohol policy developments meant that the alcohol strategy was developed in a policy context which was already being shaped by the PHRD; the origins of which are traceable back to at least 2008. As a cornerstone of the government’s alcohol policy, the PHRD had an important structuring effect on subsequent policy debates in this period. As a civil servant familiar with this process commented:

There was a well-established process that we were keen to build upon. […] There was a Public Health Responsibility Deal, which the Department of Health led on and we wanted to build on that but looking at whether there was potential for additional industry pledges in some of the areas that we had identified. […] But also for us there was a framework there in which we could build on and there were people within the industry that led on elements of that Responsibility Deal who were able to broker a degree of consensus across the industry.

The influence of the RDAN over other policy initiatives was confirmed by another civil servant in a different government department:

I suppose through the forum of the responsibility deal, civil servants saw that as their priority. [..] So, […] it’s how can we get this to work? How can we get this to deliver? The minister’s priority would be in that case the Responsibility Deal because this was a big project; a big ideology they really wanted to push. […] The thing about civil servants is […] it’s almost like they’re project managers. They’ve got a project and they want to go forward. Yeah, I mean within their code they’re impartial. Policy direction is set by ministers and then it’s their job to make it work.

### Circumscribing the policy space

By definition, co-regulatory regimes such as the PHRD are designed to develop and implement measures, which can be delivered through collaborative rather than legislative measures. The decision to pursue an approach such as the PHRD skewed the policy agenda away from regulatory measures (such as pricing policy). Public health actors involved with the RDAN indicate also that the range of policy options available for discussion within the context of the RDAN were highly circumscribed from the very outset. According to one public health actor, this reflected the privileged position of the alcohol industry in the policy’s development and their degree of engagement with policy makers before the input of public health actors was sought:

When we were first invited to engage in the Responsibility Deal, as an initiative, we did go forth with a bit of an open mind, well, this is a new scheme, by the new Government, let’s see what it’s all about. And, essentially, when we arrived at the table, it was very clear that the entire framework of the pledges and the voluntary arrangements, had already been established behind closed doors, between Government and the alcohol industry. So, we were very aware that the alcohol industry had very easy access to the Government, they had regular frequent meetings with the Government, they were in regular dialogue with the Government. And, the Government was looking very favourably on a self-regulatory, voluntary approach.

This was confirmed by another public health actor with knowledge of the establishment of the PHRD:

It was made absolutely clear to us that we could only talk about things which were in the remit of DH. And therefore we were not permitted to discuss fiscal policy, MUP or any of those. Obviously we tried to do that every meeting, but we were basically told you are not allowed to talk to us about that.

Those measures that were open for consideration, and which may have had some degree of effectiveness, were taken up by the industry only to the extent they did not undermine their commercial interests. The limits of the voluntary approach were circumscribed by divisions within the industry. For example, proposed measures on labelling products with unit content information were opposed by some manufacturers who felt their products would be adversely effected, or seen more negatively than others. For example, the RDAN was only able to agree on the labelling of cider cans, rather than all containers (including bottles), because cider producers, some of whom sell products in three-litre plastic bottles (containing 22 units) would not agree to the measure. Other interviewees, meanwhile, suggested measures proposed by industry actors were largely activities in which they were already engaged but were seeking to rebrand within the context of the RDAN rather than developing new approaches:

Sometimes what we were being presented with, was more of what the industry was doing already, rather than an opportunity to discuss perhaps collectively how we might try something different.

For some respondents it was clear that participation in the RDAN, and the narrow policy agenda this implied, was a key industry strategy for diverting attention away from more effective policy options, which they opposed, and which could not be delivered through this mechanism. This was echoed by a public health actor with knowledge of the RDAN process:

They were doing what you would expect them to do which was protect their bottom line. You know their duty is to protect shareholder value; they have not duty to protect the health of the population, that’s up to the government. So the only reason they were there was to defer or prevent effective policy. I think it was successful in doing so.

The RDAN offered an environment in which alcohol industry actors were key partners in policy-making. This undermined the ability of government to act independently because the approach centred on the co-production of policy. In the words of one civil servant with intimate knowledge of the process:

And again working with the grain of the industry, looking at where we can build on existing frameworks […]. We wanted to be almost like a critical friend really, looking at that and challenging them to go further in certain areas. Again, this is an opportunity for the industry to demonstrate how serious they were about doing some of these things. […]. We would regularly draw on bits of evidence from the police, from alcohol charities, public health practitioners etc around particular products or retail practices or promotions or whatever it might be, and ask the industry to account for those and to discuss how the industry could do things differently. That’s the form that that dialogue took.

The PHRD thus offered industry actors a highly formalised mechanism through which they were able to manage the alcohol policy environment through ongoing engagement with civil servants, who were, in turn, under pressure to deliver the policies decided upon at ministerial level. As one senior civil servant commented in relation to the industry:

It was through the responsibility deal […] they would be engaging all the time. They are sort of very good at that sort of constant sort of trying to engage with civil servants all the time, which I think has the effect of putting them at the top of civil servants’ minds. You’ve also got their engagement with ministers themselves through whatever mechanisms they use […]. It’s almost trying to tie civil servants in knots. […] sort of constantly demanding things of them, wanting answers to things. […] it’s almost as if they set challenges […], what’s happening on that? What are you going to do about that?

### An opportunity for industry

The PHRD created an institutionalised mechanism through which industry actors could make plausibly legitimate demands on key parts of the machinery of government. This represented an additional call on the time and energy of civil servants and an effective way to shape their thinking on policy issues in which industry actors have significant expertise and resources. As well as the policy diverting effects of the PHRD, it had considerable value to the industry in terms of their public relations and corporate social responsibility (CSR) agendas. Cultivating a positive image of themselves as responsible actors who contribute to society is a key component of alcohol companies’ efforts to avoid regulation [33]. As a representative from a large UK charity commented:

working in partnership with government, is beneficial from a PR perspective for the industry, you know. They would talk about being involved in it, very front and centre. You know, ‘we’re working together with government and other civil partners.’ It’s a huge benefit to an industry […] not coupled with any meaningful change in their approach to, say, on the marketing of alcohol.

The success of efforts to persuade those in government of their commitment to reducing harm potentially conferred additional legitimacy on the involvement of industry actors in policy making. For example, one civil servant involved in the RDAN process regarded industry commitment to the PHRD in terms of perceptions:

I have to say that my experience of then subsequently working with the industry was one of a genuine commitment on their part to wanting to be seen to be doing more.

For the alcohol industry, the PHRD acts as a key point of reference for industry actors in the context of policy debates. It acts as an ‘artefact’ which can be pointed to in industry documents and discourses. It has a materiality and a physicality in its meetings, documents and a virtual presence online. Partnership with government implies that industry actors are acting in a positive way, curbing the excesses of their businesses and their customers; a framing perpetuated by industry CSR materials and statements, which focus on such initiatives.

The assumption that co-regulatory arrangements are mutually beneficial to both industry and government is implicit in the underlying logic and rationale for this approach. Partnership-based, voluntary regimes and codes of practise are often discussed in ways that imply industry actors are willing to subordinate their narrow, short-term interests to a greater societal good. In the case of the RDAN it appears that companies were only willing to embrace measures that did not disadvantage them commercially or that favoured them in comparison to rival sectors and companies. This underlines the limitations, which this type of arrangement places on policy development and the conflicts of interest that they engender.

### A challenge for public health

For the alcohol industry the PHRD represented a considerable opportunity to shape policy and cultivate its image in the eyes of policy makers and the general public. For public health actors, however, it quickly became a challenge to be managed rather than an opportunity to shape policy. NGOs and health bodies faced difficult choices about whether to participate in the RDAN. This led to divisions emerging within the public health and alcohol policy communities. It was evident to some that, in the absence of further policy developments, the partnership based approach embodied by the RDAN would shape the terrain of UK alcohol policy. The conflict at the heart of alcohol policy – between effective, evidence-based measures favoured by public health and the industry favourable approach represented by the RDAN, for which any supportive evidence is absent – was resolved in practice in favour of the industry. This meant the public health actors needed to reconcile themselves to this state of affairs, and concluded at different stages of its development that they were unable to participate in the RDAN. As the representative of one NGO commented:

Because, one of the main reasons why myself and other members of the Alcohol Health Alliance boycotted the Responsibility Deal, was because a voluntary partnership with industry was being launched, in the absence of a comprehensive Alcohol Strategy, that included fiscal measures, such as minimum unit pricing. And, we were told from the very beginning that MUP was never going to be part of the Responsibility Deal. Not only because the industry would never allow it on the table but, also, they don’t have it in their power to set prices, so it would have been inappropriate for MUP to have been discussed under that umbrella.

For others in the public health community, the degree of commitment to the PHRD demonstrated by the government meant they felt obliged to participate in the RDAN despite their clear reservations about its effectiveness and the position it afforded to industry actors in policy-making. As the implementation of the policy was inevitable, they felt it was beholden to them to try to mitigate its deleterious consequences and lobby for whatever positive policy developments were possible within its remit. As one public health respondent familiar with the establishment of the RDAN commented:

But we also knew what we were dealing with a government who were committed to this route and, therefore, we felt obliged to join in and to ensure that, as far as possible, everything was evidence based and everything was properly evaluated. […] That’s what we continued to do up until the point at which the government did their U-turn on alcohol strategy because what we were told all along is that you join in with the RDAN then we will be working on this alcohol strategy, and it will be a good alcohol strategy.

Some health bodies participating in the RDAN perceived their co-operation in this initiative a quid pro quo for the introduction of pricing measures. The government’s failure to proceed with the implementation in 2013 confirmed others’ suspicions that the course of alcohol policy had already been set and that even participation in the government’s flagship policy would be unable to shape wider developments and deliver key public health objectives.

Unlike industry actors, the PHRD had the effect of limiting, rather than facilitating public health actors’ access to government. This gave many in the public health community the impression that the PHRD was essentially a partnership between government and industry to which civil society bodies were invited to add credibility, whilst being regarded as a threat to the arrangement:

So, we had meetings, we were called in to meet the Minister, Anne Milton, at the time. Just before we all walked away from the Responsibility Deal, I think her objective was to knock us into line and tell us that we had to play ball. And, at that meeting, we said, we simply cannot have action on alcohol that doesn’t include addressing price. […] We weren't given as easy access to the Ministers. The Minister only met us when we were threatening to upset the apple cart, we wouldn’t have got a regular meeting otherwise.

The existence of the PHRD structured the form and content of engagement with public health actors as well as industry. It became the default mechanism for engagement with government on health issues and necessarily focussed that engagement on the industry favourable rather than public health focussed agenda, which the PHRD institutionalised. Other channels of engagement which may have existed previously were rerouted this way. Furthermore, non-participation in a flagship government policy was seen as a hostile act by Ministers and made dialogue with them even harder. This created serious dilemmas for public health actors around the PHRD. As One NGO representative commented:

We were, at the time, still relatively unpopular amongst the Government, because we’d boycotted the Responsibility Deal. And also, there was no other vehicle for having regular contact with Government other than the Responsibility Deal, so we were excluded from that. […] so it’s almost like a take it or leave it for the NGOs, sign up to this, kind of, fundamentally flawed model, with all sorts of conflicts of interest in it, and have a voice and a channel to the Government, or be completely put by the way side. […] and, after a number of important NGOs had decided not to participate in the Responsibility Deal, we asked the Government for an alternative forum that would free from vested interest, where we could discuss policy issues. And, we did not get that, until all NGOs had resigned from the Responsibility Deal, and that was following the announcement that minimum pricing wasn’t going to happen.

For the alcohol industry, the PHRD represented not just the institutionalisation of a highly favourable policy regime, but a forum in which they could regularly engage policymakers, shape the policy agenda and steer resources away from potentially damaging policy developments. In contrast, it had damaging effects on public health actors’ ability to advocate for the policy measures which evidence indicates are most likely to reduce alcohol harm by closing off avenues of dialogue with decision makers and making health NGOs take highly public stands against a key government policy, which alienated them from the PHRD’s political sponsors.

### The power of partnership as a policy idea

The ideas of public-private partnership in policy making, and self- and co-regulatory agreements such as those enshrined in the PHRD (from here on ‘partnership’) exerted deep influence over policy actors. This was perhaps most true of policy-makers and administrators, but its effects were evident even amongst health advocates. In all these groups, to different degrees, there appeared to be an acceptance that partnership was the default approach to policy making, as the first option to be considered, before other policy measures only entered onto the agenda once partnership had been exhausted. As such, more interventionist policy measures, as indicated by the evidence base to require regulation of industry actors, are at a disadvantage from the very outset. Advocates must make the case for their adoption from a reactive position. Whilst the RDAN may represent an institutionally developed form of partnership encompassing a range of health conditions and industries across multiple policy debates, it is simply the latest in a long line of example of this approach in UK alcohol policy. As one government actor commented:

I would say, because I’ve worked on it for quite some time in [government], it’s a bit depressing how I think I saw about four cycles of voluntary initiatives on alcohol, so my feeling is the issue is for ministers and politicians of both main parties, it’s kind of a default approach to work in partnership with industry. It’s just they psychologically have tended to see it as the easier approach. If we’re not sure what to do, or if this minimum unit pricing looks a bit difficult, it’s much easier to get industry to do something.

In part, this reflects the lack of institutional memory resulting from the rotation of civil servants between posts, meaning as policy debates develop key positions may be occupied by new people who were not in place during previous iterations of the partnership arrangements. It also recognises the power of the alcohol industry in UK alcohol policy.

The government respondent cited above argued that partnership based approaches are likely to have become more appealing given the large cutbacks which have occurred within the civil service since the coalition government came to power. These cutbacks mean that producing evidence reviews and the background work needed for the development of a new policy initiative such as MUP would entail a great burden, notwithstanding scepticism amongst civil servants about partnership approaches. As one government respondent commented: “I can remember at the time thinking, well there’s no proof that this [partnership approaches like RDAN] has ever worked before.”

The hold of ideas about partnership had important effects on the public health community such that they felt an obligation to try to make it work, despite the evidence -base and the conflicts of interest they raised, meaning that it was unlikely to be effective:

As far as we were concerned we were there to show that we were trying to make this work as best we could, in the knowledge it wasn’t going to have any effect at all

This was echoed by another public health actor involved in the establishment of RDAN:

The most important thing the Responsibility Deal achieved, from my perspective, was that we tried it and that might sound a very low-level accomplishment, but it would have been very easy for industry in the absence of us trying to sit around and say, well anything could have been possible if the health sector just turned up for the discussion. […] I think that the reality is that things which will significantly reduce alcohol sales and consumption are not going to be agreed by an industry that relies on that for their profits. [….] Now we can add, because we tried that, and it didn’t.

The idea that regulatory approaches can only be considered once partnership approaches have been attempted and shown to be ineffective is evident in other sectors as well as alcohol. As a representative of an NGO concerned also with food commented on both:

But if that doesn’t work, then you have to be prepared to move to the next step, which is a more…something with sanction, with some force behind it, something compulsory.

Some public health actors and civil servants believed that ongoing adherence to partnership approaches reflected a lack of genuine political commitment to reducing alcohol related harms via reduced consumption as much as a misplaced faith in partnerships. The suspicions about the lack of genuine political commitment to effective policies to tackle harms via reduced consumption were informed by perceptions that there are risks of unpopular decision-making with adverse fiscal implications. Other explanations for the persistence of partnership-based approaches resulted from a lack of strategic thinking, and thus clarity, about the underlying policy objective to be pursued. At the same time, governments felt compelled to act, and be seen to be acting, on the issue of alcohol leading to disjointed and sub-optimal policy prescriptions. As one governmental respondent commented:

the solution is put up first, and that isn’t based on trying to solve the problem. It’s often based on more what’s acceptable politically rather than… So I think getting to that crux of what’s the problem and what do we want to achieve; [...] what policies are going to get you there?

Other government actors were strongly committed to the partnership model as an effective way of addressing alcohol harms. Such views often treated industry actors and public health actors as two different interests groups within the policy process whose views and interests need to be balanced with one another. Asked whether it would be possible to make alcohol policy without industry engagement in the process, one governmental actor suggested this would be suboptimal and limit policy options open to government:

My only observation of that would be I think it would be quite limited because really all you are left with then doing is regulating. Now regulation has its merits and there is a place for regulation and there is wonderful things you can do with regulation and we have seen examples of that and tobacco is a good example of that. We can regulate, we can restrict the sale of products, we can mandate certain types of labelling, we can do all those things, we can tax, all these things that are the traditional toolbox of government and that’s fine. There is always that place for regulation. But if you want to be a bit more imaginative in policy making, […] you have to then look at other solutions.

## Discussion

The emergence of the PHRD is vital to understanding wider developments in UK alcohol policy since 2010, particularly on alcohol pricing policy [20]. This is in keeping with previous studies of alcohol industry strategies in the UK [5, 13, 14, 21, 36–38], which identify industry lobbying and framing activities designed to shift policy debates towards self- and co-regulatory regimes that have little supporting evidence [4]. In addition, it adds to the wider literature on corporate political strategy in other sectors [25–28]. Their support for the PHRD reflects the determination of the industry to avoid ‘whole population’ measures such as MUP and other forms of regulation coming onto the policy agenda, which are viewed as detrimental to their interests.

It is important to give due regard to various limitations of this study. The interviews were conducted some years after the events being discussed. There is obvious potential for problems with recall, making triangulation of different accounts essential. These accounts also should not be regarded as purporting to offer a definitive oral history of the RDAN; rather they are better read as plausible narratives about events, and activities around them and their inter-relationships, developed in good faith by researchers on the basis of expert testimony by key actors with access to, and knowledge of, the relevant policy process. The data analysed do not include interviews with industry actors, so it is appropriate to question the validity of the perceptions of the various actor types, particularly in connection with study findings on the industry itself. Similarly, how far is it possible that interviewees were providing accounts that they believed we wished to hear? We suggest that a careful reading of the findings with these concerns in mind will reveal that there is little in the material presented that could be regarded as being contentious, and we also recommend attention to the prominence we have given to findings on public health as well as industry actors.

The significance of the PHRD for the alcohol industry extended beyond simply providing a device to manage policy-making and having additional rhetorical importance as part of industry CSR strategies. The PHRD structured wider policy debates in this period and the forms of engagement between government, the industry and public health actors. It involved a hitherto unparalleled institutionalisation of industry involvement in alcohol policy making. As a consequence, it fostered divisions within the public health community about how to respond to a hostile policy environment and whether to participate in the PHRD. In declining to participate in, or withdrawing from, the government’s key public health policy initiative, health actors input into policy-making and access to key decision makers was diminished at precisely the same time that industry influence was secured. For the alcohol industry, therefore, this was a double victory.

The decision to pursue the PHRD in advance of the government alcohol strategy made it the key political priority for civil servants in the DH and the Home Office; the main government departments tasked with delivering alcohol policy. Other policy initiatives were marginalised in this context. MUP, for instance, came onto the Westminster policy agenda, and into the alcohol strategy, via the initiatives within the Cabinet Office, with DH and Home Office unaware of its planned inclusion until the very final stages of the process. This reflects the alternative (RDAN-focussed) policy agendas established within these key ministries and the lack of political ownership evident for the most effective policy proposal emerging in UK alcohol policy in this period.

The formation of the PHC, and its development into the PHRD, demonstrate the effective ways in which industry actors are able to influence policy through long-term engagement and relationship building with key policy-makers and the delivery of policy goods for government, as well as the value of engaging with political parties whilst in opposition [32, 38]. The adoption of partnership-based approaches and co-regulatory regimes, in the PHRD, systematically skewed the incoming government’s policy agenda towards the least effective forms of policy interventions from the very outset and created a path dependency for subsequent policy development.

Despite the problems examined above, and widely articulated within the public health community, the idea of co-operation and engagement with industry ‘partners’ retains a strong hold over governmental actors and even many within the public health sector. This is evident most recently in the controversial decision of Public Health England to partner with industry body Drinkaware for the delivery of public messaging campaigns [45]. However, the prevalence and the enduring appeal of partnership-based approaches in health is not limited to alcohol or to the UK. As such, the findings here are of relevance to policy makers and advocates working on other issues and in other contexts. This article identifies potential ways of thinking about research questions and methods for answering them in relation to the policy structuring effects of co-regulatory regimes. These insights could be applied within, for instance, the food and beverage industry, in other national settings, at local and regional level and in supra-national settings such as the EU and WHO, as well as to public health actors, as has been done here.

When faced with decisions on how to design and implement policy measures, particularly those which address major societal and public health challenges, governments could reverse the current logic. While regulation is currently considered only where voluntary approaches and partnership have failed, policy makers should instead start with the research evidence for the most effective responses. Partnership could be restricted to particular stages or aspects of the policy process, for example in respect of the implementation of policy decisions made in the public interest. Strong protections against the influence of vested-interests would still need to be adopted. Whilst partnership approaches may imply the ability to mitigate some of harm done by alcohol, as they have developed in UK alcohol policy, they undermine the very causes they seek to further.

## Conclusion

Self- and co-regulatory regimes, based on partnership approaches between government and industry have been extensively criticised within alcohol policy and within the wider field of public health on the basis that such approaches are ineffective and ignore the prevailing research evidence on effective policies to address harms. Drawing on insights from the corporate actors and policy studies literature, this article moves beyond these critiques to demonstrate that the effects of co-regulation in UK alcohol policy extend beyond just diverting time and resources away from particular effective alternatives such as price increases and/or restrictions on availability and marketing. The PHRD had the effect of structuring the entire policy terrain, dictating both the content and form of policy debates. It circumscribed the policy space and institutionalised lines of access and influence for industry actors. At the same time, it presented public health and civil society actors with a dilemma about whether to participate in structures riven with such conflicts of interest. This led to both disagreements and divisions within the public health sector and marginalised these bodies from policy debates, which were channelled through the RDAN. Whilst this study focuses on England, its findings are of wider relevance and offer insights which can inform analyses and critiques of similar attempts to implement co-regulatory regimes in other policy settings. This is of vital importance given the ‘stickiness’ and intuitive attractiveness of partnership as a governance principle and the primacy afforded to it in many policy contexts.

## Data Availability

The datasets generated and/or analysed during the current study are not publicly available due to the fact we offered interview respondents anonymity in order to participate in the study. No transcripts can, therefore, be made available to third parties within the terms of the informed consent given by interviewees.

## Abbreviations

COI: Conflicts of interest
CSR: Corporate social responsibility
DH: The Department of Health
MUP: Minimum unit pricing
NCDs: Non-communicable diseases
PHC: Public Health Commission
PHRD: Public Health Responsibility Deal
RDAN: Responsibility Deal Alcohol Network
